# Local Treatment of Burns with Cell-Based Therapies Tested in Clinical Studies

**DOI:** 10.3390/jcm10030396

**Published:** 2021-01-21

**Authors:** Anna Paulina Domaszewska-Szostek, Marta Olga Krzyżanowska, Anna Maria Czarnecka, Maria Siemionow

**Affiliations:** 1 Department of Human Epigenetics, Mossakowski Medical Research Centre, Polish Academy of Sciences, 5 Pawińskiego St, 02-106 Warsaw, Poland; 2 Division of Ophthalmology and Optometry, Department of Ophthalmology, Collegium Medicum, Nicolaus Copernicus University, 85-067 Bydgoszcz, Poland; marta.krzyz@gmail.com; 3 Department of Neurotoxicology, Mossakowski Medical Research Centre, Polish Academy of Sciences, 5 Pawińskiego Street, 02-106 Warsaw, Poland; aczarnecka@imdik.pan.pl; 4 Department of Orthopaedics MC 944, University of Illinois at Chicago, 900 South Ashland Avenue, 3356 MCBRB, Chicago, IL 60607, USA; siemiom@uic.edu

**Keywords:** burns, radiation burns, keratinocytes, fibroblasts, mesenchymal stem cells, adipose tissue

## Abstract

Effective wound management is an important determinant of the survival and prognosis of patients with severe burns. Thus, novel techniques for timely and full closure of full-thickness burn wounds are urgently needed. The purpose of this review is to present the current state of knowledge on the local treatment of burn wounds (distinguishing radiation injury from other types of burns) with the application of cellular therapies conducted in clinical studies. PubMed search engine and ClinicalTrials.gov were used to analyze the available data. The analysis covered 49 articles, assessing the use of keratinocytes (30), keratinocytes and fibroblasts (6), fibroblasts (2), bone marrow-derived cells (8), and adipose tissue cells (3). Studies on the cell-based products that are commercially available (Epicel^®^, Keraheal™, ReCell^®^, JACE, Biobrane^®^) were also included, with the majority of reports found on autologous and allogeneic keratinocytes. Promising data demonstrate the effectiveness of various cell-based therapies; however, there are still scientific and technical issues that need to be solved before cell therapies become standard of care. Further evidence is required to demonstrate the clinical efficacy and safety of cell-based therapies in burns. In particular, comparative studies with long-term follow-up are critical.

## 1. Background

Burns represent a substantial public health problem worldwide. According to the World Health Organization, there are approximately 265,000 deaths each year due to fires, electric burns, and chemical substances [1]. Furthermore, over 96% of fatal fire-related burns occur in low- and middle-income countries [1]. Thermal burns are the most common type of burn injuries, making up about 86% of the burned patients requiring burn center admission [2]. The burn depth is proportional to the temperature of the causative agent and the contact time length [3]. Effective wound management is a major challenge and an important determinant in patients′ survival and prognosis with severe burns.

Burn wounds are characterized by loss of progenitor cell population that is necessary for the epidermis and dermis regeneration [4]. Severe burns would benefit from cell therapy by enhancing wound healing, replacement, and regeneration of damaged skin. A significant challenge is incorporating skin appendages, reducing fibrosis and inflammation [5]. Furthermore, by destroying the epidermis, the likelihood of bacterial infection increases [6].

**Figure 1:**
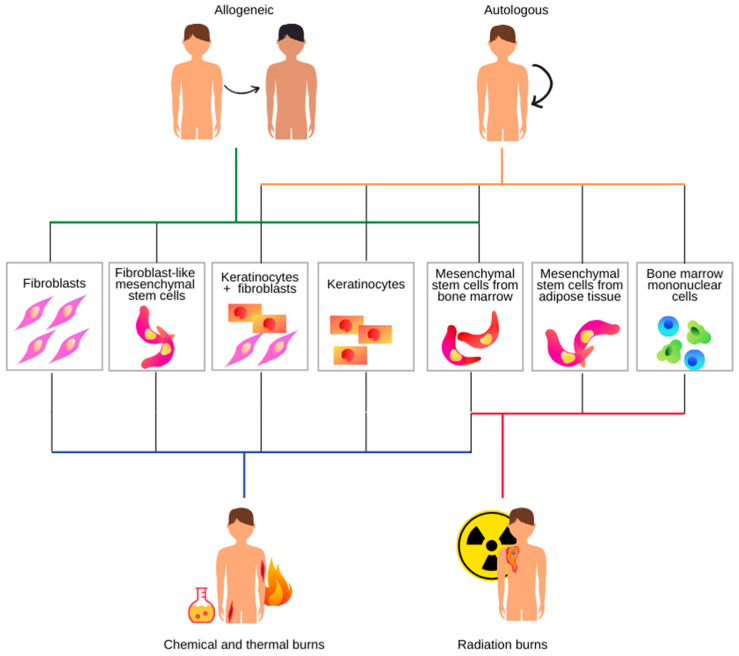
Cell therapy strategies for burn wound healing.

We decided to separate cell therapy application in radiation burns from other types of burns in our review (Figure 1). Although a radiation injury is often referred to as a “burn”, the development of the injury is considerably different from that of a thermal or chemical burn [7]. Unlike chemical or thermal skin injuries, the lesions caused by radiation exposures do not appear for hours to days following exposure [8]. Possible harmful side effects of radiotherapy to surrounding healthy tissues often lead to necrosis [9]. Irradiation can also hinder wound healing, leading to chronic ulceration. It impairs proliferation, differentiation, and secretion of extracellular matrix proteins and growth factors. Besides, the presence of reactive oxygen species can lead to dysregulated myofibroblast production, resulting in abnormal collagen production. Wound healing impairment by inhibiting inflammatory cells and angiogenesis is known as radiation-induced fibrosis, a characteristic feature of delayed radiation injury [10]. To date, there is no standardized treatment for chronic radiation-induced wounds. Due to the observed constant inflammation, both at the site of injury and systemically, skin transplant failures occur. Thermal and radiation burns differ in their inflammatory response. After radiation burns, recurrent inflammation occurs, leading to erythema at the exposure site and finally necrotic wounds [11,12].

Surgical debridement followed by split-thickness skin grafting (STSG) is a standard therapy for burns. One of the biggest problems with severe burn patients is the limitation of available donor skin sites for surgical treatment, especially in cases with over 50% of total body surface area (TBSA). Thus, the technique of meshed STSG allows expanding skin grafts to a much larger size [13,14,15]. However, when the expansion is over 1:6 of meshed grafts, there is a lower re-epithelialization rate and a significant decrease in survival rate [13]. Moreover, STSG increases the wound’s total surface, leading to higher water and electrolyte loss from the patient’s body [16]. Another issue is donor site hypertrophic scar and contracture, especially in children due to their physical growth [17]. Re-epithelialization is crucial in the burn wound treatment, and there are many different methods of delivering skin cells to the wound bed [13,18]. Many efforts have been made towards autologous and allogeneic cell-based therapies and skin substitutes, both as monotherapy and as a part of combined treatment.

This review aims to overview the up-to-date local treatment of burns with cellular therapies based on the published data from 1983 up to 2020. We discussed the results of clinical studies only. Practical options for future therapeutic applications of cell therapies for burns treatment and ongoing challenges associated with burn injuries are finally considered. In our previous review concerning the use of cell-based therapies in non-healing wounds, we presented the characteristics of all cell types; therefore, we did not repeat this information it in this review [19].

## 2. Experimental Section

### Study Selection

Using Pubmed search engine and ClinicalTrials.gov, we analyzed the available data concerning the use of human keratinocytes, fibroblasts, bone marrow cells, adipose tissue cells, as well as cell-based products available on the market like Epicel^®^, Keraheal™, ReCell^®^, and JACE^®^. We have excluded all preclinical (animal) studies. A systematic literature search was conducted from 1983 to 2020 using the following terms “burns” OR “radiation burns” combined with “keratinocytes” OR “fibroblasts” OR “mesenchymal stem cells” OR “adipose tissue”. Only articles published in peer-reviewed scientific journals were included in the analysis. The analysis covered 49 English-language articles, assessing the use of keratinocytes (30), keratinocytes and fibroblasts (6), fibroblasts (2), bone marrow-derived cells (8), and adipose tissue cells (3). The patients’ characteristics include the degree of burn, age, and the length of follow-up.

## 3. Results

### 3.1. Clinical Research and Applications of Cell-Based Therapies for Burn Wound Healing

#### 3.1.1. Autologous Keratinocytes

Autologous keratinocytes may be administered as a cultured and non-cultured cell suspension in the spray device, single-cell suspension, and in the form of cultured epithelial sheets [15]. Cultured epithelial autograft (CEA) is prepared as a sheet (25 or 50 cm^2^) consisting of isolated and cultured keratinocytes fixed on petrolatum gauze [15,20]. Approximately 2–3 weeks are required to prepare a confluent sheet [21]. CEA is efficient for extensive skin burns when available healthy skin is insufficient, but it is high cost and the lack of dermal substrates limits their applicability. Another disadvantage of this method is a long-term culturing period, which extends the time between biopsy and grafting. Moreover, cell culturing has other difficulties such as lack of adherence and wound contracture [22,23].

One of the pioneers in this field, Gallico et al., demonstrated that cultured autologous epithelium could be used to generate permanent epidermis on half or more of the TBSA [24]. The use of the CEA in the treatment of deep second-and third-degree burn wounds was described by Teepe et al. The authors observed that the wounds excised at an early stage showed a significantly better graft take than non-excised chronic wounds that were grafted at a later stage. The regenerated skin was smooth and pliable. Moreover, scars showed less hypertrophic formation in comparison with meshed grafts. The authors showed an inverse correlation between the graft take and the patient’s age [25]. This correlation was not confirmed by the multicenter study of Odessey et al., demonstrating that patient’s age, burn size, and extent of full-thickness injury did not significantly affect the graft’s take. The average final take was around 60%, and 22% of patients achieved a final take of ≥ 90% [26]. Another advantage of CEA transplantation in burn patients is reduced mortality. In a study by Munster et al., there was a decline in mortality from 48% to 14% [27]. Increased collagen deposition, decreased stromal cellularity, and significant effect on connective tissue phenotype and dermal neogenesis after CEA transplantation were observed by Compton et al. on a group of pediatric patients [28]. CEA may also be a part of a combined burn wounds treatment. A 15-year-retrospective study by Auxenfans et al. revealed that it allows rapid healing of STSG donor sites and deep second-degree burns, due to the decreased wound surface and stimulated healing of the remaining wound [29]. Chrapusta et al. conducted a study on children with significantly shorter healing time when STSG and cultured autologous keratinocytes were applied in one stage [30]. In 2015 Matsumura et al. published studies on CEA treatment of patients with severe burn wounds. CEA, whose manufacturing period was between 22 and 30 days, contributed to wound closure and patients’ survival [31]. In a retrospective study by Wood et al., patients were treated with CEA in the form of a sheet, cell suspension (CellSpray), or both. After an average of 10.6 days, cell suspension could be administered compared with 25 days for cultured sheet grafts due to the faster time of the pre-confluent stage cell culture [32]. Kym et al. observed no clinical differences between the sheet and spray-type of CEA; both resulted in significantly higher patient survival than the non-CEA group [33]. Another retrospective study was published by Cirodde et al. Favorable outcome was most often associated with young age and a small number of infectious complications [34]. Chua et al. published a 12-year retrospective review of patients who received CEA. The authors compared the outcomes after STSG and micrografting, both followed by the CEA application, and observed that a significantly lower amount of skin allografts was needed in the micrografting group [35]. The comparison of CEA, Cuono’s method, and CEA combined with STSG were analyzed in a study by Lo et al. The Cuono’s method is based on the two-stage procedure. A cadaver skin allograft is grafted on a wound. After 2–3 weeks, the cadaver epidermis is removed, and CEA is applied. Sites treated with either Cuono method or CEA with initial take rates < 60% did not heal. Moreover, the highest take rate was achieved when CEA was combined with STSG [36]. The most extensive study on CEA to date was performed by Hickerson et al. The results of this analysis were compared to the patients’ outcome with comparable burns, reported in the National Burn Repository. This study’s main conclusion was that when CEA was used with STSG, the survival rate increased [37]. Clinical applications of autologous keratinocytes in burns are summarized in Table 1.

#### 3.1.2. Products Based on Autologous Keratinocytes

- Epicel^®^

Epicel^®^ is a wound dressing composed of the patient’s autologous, proliferative keratinocytes sheets. FDA-approved indication for use in adult and pediatric patients with deep dermal or full-thickness burns comprises a total body surface area greater than or equal to 30%. It may be used in conjunction with split-thickness autografts, or alone in patients for whom split-thickness autografts may not be an option due to the severity and extent of their burns [38]. This CEA serves as a successful permanent burn coverage in severely traumatized patients. According to the FDA, Epicel^®^ ranges from 2 to 8 cell layers thick and measures approximately 50 cm^2^ [38]. Age is one of the factors determining the CEA’s take, as reported by Carsin et al. in a five-year, single-center study. Those younger than 15 years old presented the highest initial and final Epicel^®^ CEA take (82.28% and 85.27%, respectively). More extensive burns tended to occur in the younger population, which contributed to these results. Surprisingly, no correlation between the take and burn wound size was observed. The authors stated that Epicel^®^ CEA appears to have a high beneficial value in managing burns covering over > 60% of TBSA [39].

- Keraheal™

Keratinocyte spray suspension is the next method for delivering epidermal cells to the wound bed. Keraheal™ is one of the products available on the market (Biosolution Co. Ltd, Seoul, Korea). Unlike the conventional sheet type, it contains mainly non-differentiated pre-confluent cells. According to the manufacturer, this spray-type autologous keratinocyte therapy is indicated for deep second-degree burns covering more than 30% of TBSA and in third-degree burns—more than 10% of TBSA [40]. Keraheal™ improved scars′ quality in severely burned patients and was effective in saving lives [12]. According to Yim et al., Keraheal™ requires a lower number of cells to culture and shorter culturing time in comparison to the sheet type. In studies of Keraheal™ combined with meshed grafts, the CEA’s take rate after four weeks was 100% in Yim’s and 68% in Lee’s study [13,41].

- ReCell^®^

ReCell^®^ is another product applied via spray (Clinical Cell Culture, Cambridge, UK). This system uses rapid, autologous cell harvesting, processing, and delivery technology. A small sample of the patient′s skin is obtained to isolate keratinocytes, fibroblasts, and melanocytes that are sprayed over the burn wound by a special nozzle [42]. FDA-approved indication for the treatment of acute thermal burn wounds in patients 18 years of age and older. An appropriately licensed healthcare professional uses the RECELL^®^ Device at the patient’s point-of-care to prepare autologous Regenerative Epidermal Suspension (RES™) for direct application to acute partial-thickness thermal burn wounds or application in combination with meshed autografting for acute full-thickness thermal burn wounds [43]. A randomized trial comparing results obtained with the ReCell^®^ autologous cell harvesting (ACH) system and the classic skin grafting for epidermal replacement in the deep partial-thickness burns was performed by Gravante et al. Their study revealed that skin grafting was faster than ReCell^®^, but ReCell^®^ biopsy areas and post-operative pain were smaller than in traditional grafting [44]. Not only adults received ReCell^®^, Wood et al. performed a randomized controlled pilot study on pediatric patients with partial-thickness scald injury. They tested if the addition of ReCell^®^ to the Biobrane^®^ synthetic wound dressing gave better results and compared them to the standard treatment—skin grafting [45]. According to the manufacturer, Biobrane^®^ is composed of a silicone membrane bonded to a nylon mesh. Peptides from porcine dermal collagen have been connected to the nylon membrane form a flexible and conformable composite dressing. Biobrane remained attached to superficial partial-thickness burn wounds, donor sites, and excised burn wounds with or without meshed autografts [46].

In the Wood et al. study, by day 21 after burn, 100% of patients receiving Biobrane^®^ and ReCell^®^ healed, 97.7% receiving Biobrane^®^, and 90.1% in the standard treatment group. According to the authors, the best outcomes can be obtained when debridement, followed by Biobrane^®^ with or without ReCell^®^, is performed within four days after-burn. It leads to decreased healing time and requires fewer dressing changes. Moreover, it is less painful [45]. In another study by Sood et al., the effects of ReCell^®^ treatment were compared to the effects after a meshed STSG. Eight patients had 100% take with both treatments, and two patients had significant non-take and graft loss. Patients benefited from the ReCell^®^ therapy having a decreased donor site size and comparable outcomes with meshed STSG treatment [47].

- JACE ^®^

JACE^®^ is a Green-type CEA, an epidermal cell sheet supplied in cultured autologous epidermis produced from keratinocytes for treatment of severe, extensive burns. It allows obtaining cells from a small area of the patient’s tissue. These sheets are grafted onto the wound surface with preserved dermis for the closure of the wound via engraftment and epithelialization [48]. JACE^®^ is indicated for patients with a deep-dermal or full-thickness burn wound when sufficient donor sites for autologous skin grafts are not available, and the burn area is 30% or more of the TBSA. After skin grafting, a cultured epidermal cell sheet is applied onto the reconstructed dermis [49]. The results from a 6-year multicenter study of JACE^®^ were published by Matsumura et al. The authors demonstrated a 66% take rate at four weeks after grafting and found that the JACE^®^ application contributed to patient survival up to seven weeks after burn [50]. Similar results were obtained by Hayashi et al., who used a combination of JACE^®^ CEA and STSG or meshed split-thickness dermis graft, de-epithelialized STSG. A meshed dermis graft required more healing time than STSG, but it enabled covering a burn wound by collecting tissue from only a small donor site. The skin graft taken at four post-operative weeks was between 85 and 95% [48]. Clinical applications of products based on autologous keratinocytes in burns are summarized in Table 1.

#### 3.1.3. Autologous Engineered Skin (Keratinocytes and Fibroblasts)

Hansbrough et al. developed procedures for establishing confluent, stratified layers of cultured, autologous keratinocytes on a modified collagen-glycosaminoglycan membrane containing autologous fibroblasts. These grafts were transferred onto the areas of full-thickness burn wounds. It took up to 9 days to form the basement membrane. According to the authors, this technique offers a significant advance in extensively burned patients’ care and can also provide skin for reconstructive surgeries [51]. Boyce et al. investigated cultured skin substitutes (CSS) consisting of autologous cultured keratinocytes and fibroblasts. The cells were attached to collagen-based sponges prepared from STSG. When CSS is used, donor skin can be spared, and the mesh ratio for autografts needed for coverage of the remaining, not-covered burn could be reduced to 1:2 or less. Reduced mesh ratio autografts guaranteed faster healing and reduced scarring [52].

TESE is a tissue-engineered skin equivalent developed by Takami et al. It comprises autologous cultured keratinocytes, fibroblasts, and a decellularized allogeneic dermis and requires three weeks of processing. After this time, it was transplanted to the third-degree burn wounds. The authors observed 96% of graft survival. TESE′s histological characteristics are similar to normal human STSGS. Therefore, it can be used for permanent repair of full-thickness skin defects [53]. Clinical applications of autologous keratinocytes and fibroblasts in burns are summarized in Table 1.

#### 3.1.4. Allogeneic Keratinocytes

Hefton et al. observed that burn wounds grafted with cultured allogeneic epidermal cells healed within three days and remained healthy for the nine months of observation. Based on these findings, the authors stated that allografts might serve as alternative biological dressings, or grafts, for deep second-degree burn wounds. They accelerate healing and reduce the need for STSG [54]. The same conclusion was drawn by Madden et al. where cultured allogeneic epidermal cells gave similar results to the autografts in second-degree burns and were not successful in third-degree ones [55]. Faster epithelialization of the wounds was also confirmed by Rivas-Torres et al. Healing time was reduced by 37.8% when treated with cultured epidermal allografts. Moreover, the authors observed that allografted sites were less erythematous than skin grafts, which served as a treatment control [56]. Similar results were obtained after transplantation of frozen cultured human allogeneic epidermal sheets in deep and superficial partial-thickness burns. Alvarez-Diaz et al. observed that deep partial-thickness burns treated with cultured allogeneic epidermal sheets healed in an average of 5.6 days versus 12.2 days in the control group [57]. Cryopreserved cultured epidermal allografts in pediatric patients were studied by Yanaga et al. Not only early wound closure and prevention of hypertrophic scar formation but also the decrease in graft cell viability were observed. The sex-determining region Y (SRY) gene could be detected only for 2–4 weeks after cell transplantation [17]. It proves that allograft take is not permanent, and allogeneic cells are replaced by autologous keratinocytes. Haslik et al. analyzed the long-term results of dermal hand burns covered with cryopreserved allogeneic keratinocyte sheet grafts. The authors compared these keratinocytes to autologous STSG and observed no statistically significant differences. The use of allogeneic keratinocytes for the coverage is appropriate to preserve skin grafts for full-thickness areas. Because of high costs and qualified staff requirements, in the presence of sufficient donor sites, the usage of skin grafts for the application in hand burns should be the first choice of treatment [58].

On the other hand, 15 years retrospective study of Auxenfans et al. revealed that cultured allogeneic keratinocytes (CAlloK) facilitate healing of STSG donor-sites as well as deep second-degree burns. CAlloK secrets growth factors and cytokines stimulating the proliferation of host keratinocytes in both acute and chronic wounds. Due to the storage options and availability, they can serve as temporary coverage. Transplantation of CAlloK in deep dermal burns when there is a lack of donor sites may replace the use of STSG [14].

There is a product—KeraHeal-Allo™—which was investigated in phase 1 and 2 clinical trials. It is a thermosensitive hydrogel-type allogeneic keratinocyte therapy (Biosolutions Co., Ltd) to promote the reepithelialization of deep 2nd degree burns. No significant adverse reactions have been observed yet [59]. Clinical applications of allogeneic keratinocytes in burns are summarized in Table 2.

#### 3.1.5. Allogeneic Keratinocytes and Fibroblasts

Apligraf^®^ is a living, bi-layered cell-based product approved by the US. Food and Drug Administration (FDA) to heal diabetic foot ulcers and venous leg ulcers [60]. The lower dermal layer combines bovine type 1 collagen and human fibroblasts, which produce additional matrix proteins. The upper epidermal layer is formed by promoting human keratinocytes. Apligraf^®^ does not contain melanocytes, Langerhans′ cells, macrophages, lymphocytes, or other structures such as blood vessels, hair follicles, or sweat glands [61]. Waymack et al. placed Apligraf^®^ over a meshed autologous STSG over excised burn wounds. There was no difference in taking autograft in the presence or absence of Apligraf^®^. On the other hand, they demonstrated the cosmetic and functional advantages of Apligraf^®^ when applied over meshed autograft [62]. Hu et al. 2006 USA evaluated the persistence of Apligraf^®^ by DNA detection. After four weeks, it could be found only in the minority of patients [63].

Another product consisting of allogeneic keratinocytes and fibroblasts is OrCel™. It is composed of a porous collagen sponge containing co-cultured allogeneic donor epidermal keratinocytes and dermal fibroblasts from human neonatal foreskin tissue [64]. Still et al. compared Biobrane-L^®^ dressing with OrCel™ in facilitating wound closure in burn patients. The authors demonstrated that wound healing is faster after OrCel™ treatment. They explained that this effect is due to the combination of collagen sponge and cytokines and growth factors produced by the proliferating keratinocyte and fibroblast. OrCel™ sites also exhibited reduced scarring [65]. Clinical applications of allogeneic keratinocytes and fibroblasts in burns are summarized in Table 2.

#### 3.1.6. Allogeneic Fibroblasts

Cultured dermal substitute (CDS) is composed of fibroblasts seeded on a porous matrix of hyaluronic acid and collagen. Kashiwa et al. evaluated CDS as a biological dressing for highly extended mesh auto-skin grafting. When applied onto the 6-fold extended auto-skin graft, it produces growth factors and extracellular matrix components, promoting the tissue granulation and epithelialization of the skin [66]. Moravvej et al. cultured allogeneic fibroblasts on a combination of silicone, glycosaminoglycan, and autologous mesh grafts. The authors observed that allogeneic fibroblasts grafted on meshed STSG might be useful for third-degree burn wounds treatment. Furthermore, it requires less autologous skin, which is a valuable advantage in extensive burns. Healing time and scar formation compared to conventional therapy is reduced, but after 1-year, there were no differences between these two groups [67]. Clinical applications of allogeneic fibroblasts in burns are summarized in Table 2.

#### 3.1.7. Mesenchymal Stem Cells

Rasulov et al. applied allogeneic fibroblast-like bone marrow mesenchymal stem cells (BMSCs) onto deep thermal burn surfaces. The high tempo of wound regeneration in the presence of active neoangiogenesis was observed [68]. Moreover, the regeneration of sweat glands after deep burns is a significant clinical problem. Sheng et al. used BMSCs to acquire the phenotype of sweat gland cells in vitro. Twelve months after successful cell transplantation, the recovery of perspiration function in all the BMSC transplanted areas was observed. The authors emphasized that the success of the sweat gland regeneration depends not only on BMSCs but also on the surgical technique. The cells need to be covered with a decellularized allogeneic dermal matrix with laser-punched holes, granulated autologous skin grafting, and with allogeneic skin [69]. In another study, autologous transplantation of BMSCs in association with STSG was proved to prevent the skin graft contraction when combined with the injection into the sites of STSG [70]. Mansilla et al. studied cadaveric BMSC in a patient suffering an extensive skin burn. After two courses of cell transplantation and too slow epithelialization, autologous meshed skin was grafted. The skin healed completely without retractions. Limited hair regrowth was observed in the areas of BMSCs transplantation [71]. A prospective comparative study to evaluate the regenerative potential of BMSCs, and umbilical cord blood-derived mesenchymal stem cells (UC-MSCs) versus conventional early excision and grafting was performed by Wael Abo-Elkhei et al. The authors observed a significant improvement of healing both in the BMSC and UC-MSC application, with no significant difference between treatments [72]. Clinical applications of mesenchymal stem cells in burns are summarized in Table 3.

### 3.2. Treatment of Radiation Burns with Cell Therapies

Successful soft tissue reconstruction and absence of necrotic tissue with no recurrence of the lesion at 8-month follow-up was reported by Bey et al. Both surgical procedures and BMSCs therapy were performed on a 32-year-old man with a severe radiation burn of skin and underlying tissues in 3 approaches. Standard thermal burn treatment included a dermal substitute graft, but no improvement was observed. The second approach was based on muscle flap surgery and three local MSC administrations. Lack of complete healing led to the third approach—two local BMSC administrations. It resulted in a stable reconstruction of the soft tissue and complete pain relief. After BMSCs administration, a decrease of blood C-reactive protein (CRP) levels was noted, leading to the conclusion that BMSCs have an anti-inflammatory effect and accelerate the healing process [73]. Portas et al. confirmed these results. They reported the use of human cadaveric BMSCs in a patient with a radiation-induced skin lesion. The patient received three BMSCs administrations, and after each application, the CRP level decreased significantly [74]. The combination of physical therapy, surgery and local administrations of autologous BMSCs was presented in a case report by Lataillade et al. After BMSCs transplantation, almost complete healing was achieved within a month [75].

Attempts have also been made to use Adipose-Derived Stem Cells (ADSCs) in burn wounds treatment. Akita et al. treated patients with chronic radiation injuries with ADSCs and basic fibroblast growth factor (bFGF) sprayed over the radiation burn. The artificial dermis served as a scaffold. One part of the ADSCs was injected in the wound bed and margins; the other was placed over the artificial dermis. After bFGF application on a debrided tissue, increased angiogenesis and wound healing was observed. Fully regenerated tissue was seen during the 1.5-year follow-up, proving that ADSCs can be used to successfully treat the radiated wounds [76]. Moreover, beneficial effects have been reported from the transplant of lipoaspirates containing adipose-derived stem cells into wounds caused by radiotherapy [77]. Recently, a new technique has been developed—wound treatment with the cryopreserved placental membrane (vLPM). vLPM contains an extracellular matrix, growth factors, endogenous neonatal MSCs, fibroblasts, and epithelial cells of the native tissue. Regulski et al. published a case report of a 73-year-old patient with radiation necrosis. Since the wound was not qualified for surgical closure, 12 courses of vLPM were applied. The complete closure of the wound was observed at day 98 [12]. Clinical applications of cell-based therapies in radiation burns are summarized in Table 4.

## 4. Summary and Perspectives

While there have been a number of reports published on cell therapy for wound healing, clinically available therapies are still limited. In the last decade, several literature reviews have discussed the implications of cell transplantation in burn treatment [11,15,78,79,80,81,82,83]. The objective of our study was to update these findings.

The gathered data of cell-based therapies applications in burns confirms encouraging results and alternatives to standard care. In general, scientific evidence suggests that all presented bioengineered skin substitutes are safe. However, each of the presented strategies has its limitations and disadvantages. Moreover, caveats inherent with the clinical evidence covered in this report include differences in techniques measuring wound healing time and closure, small study groups, no information on how the recipient’s general health affects cell transplant acceptance. It is also difficult to conclude because burns vary significantly in depth, size, and the causing factor.

According to a multicenter experience with the treatment of partial and full skin thickness burns, the cultured autologous and allogeneic epidermis can be frozen and remains viable if stored in a skin bank [18]. There is no doubt that in comparison to the STSG, the culturing, storage, and use of keratinocytes (most studied cells) is associated with higher costs and institutional demands. The beneficial effect of their use has been demonstrated in several publications on burns in children, facial burns, donor sites, and in combination with mesh autologous skin grafts [84]. Unfortunately, a perfect technique does not exist. Despite the significant advantage of immunological safety, there are challenges with CEAs due to the long-term culture, insufficient donor site for extensive burn, and possible infections, as well as the high cost of this technique [4].

The use of isolated keratinocytes may represent an alternative therapy to CEA. The culturing time is much shorter, and the transplanted cells are not differentiated so that they may proliferate in the recipient after transplantation [15]. Therefore, due to the storage options and availability, they can serve as temporary coverage. Allogeneic keratinocytes are free of Langerhans cells and leukocytes—cells expressing major histocompatibility complex (MHC) class II, which results in a low probability of transplant rejection. Allogeneic keratinocytes do not remain in the transplant permanently and are replaced with the recipient cells [85]. Moreover, the area of skin serving as a source of keratinocytes isolation is crucial due to the number of epidermal stem cells and their proliferation potential [86].

As reported by Yanaga et al., cryopreserved cultured epidermal allografts have several advantages such as availability, the possibility of repeated treatments, enhanced wound closure, and does not require a donor’s presence [17]. In the case of severe, extensive burns, allogeneic fibroblasts grafted on meshed STSG should be considered [67]. It might be a useful method for third-degree burn wounds treatment. Furthermore, it requires less autologous skin, which is a valuable advantage. Alternatively, allogeneic fibroblast-like bone marrow mesenchymal stem cells (BMSCs) should be applied. The high tempo of deep thermal burn wound regeneration in the presence of active neoangiogenesis was observed [68]. On the other hand, it should be kept in mind that in developing countries, synthetic skin substitutes are either not available or are very expensive.

Although there is currently no significant scientific evidence suggesting that fat transplantation in acute burn wounds facilitates wound healing and improves subsequent scars, this therapeutic approach should be mentioned as a further perspective. It was confirmed that in burns, fat helps modify scar tissue by increasing vascularity and new collagen formation and deposition [87]. Several studies reported improvements in skin texture, thickness, color, and patient satisfaction [88]. Moreover, autologous stem cells from the adipose tissue of surgically debrided burned skin seem to be a promising idea. Rodney K. Chan et al. isolated these cells and proved that they could differentiate into epithelial, dermal, and hypodermal layers [89]. According to the authors, these results indicate that stem cells isolated from debrided skin can be used as a single autologous cell source to develop a vascularized skin construct without culture or addition of exogenous growth factors. This technique may provide an alternative approach for cutaneous coverage after extensive burn injuries. A very recent case study demonstrated that the adipose-derived stromal cells seeded onto a collagen-based matrix, Integra^®^DRT exhibit valuable properties that may improve post-excision wound healing and facilitate skin regeneration without scars [90]. Unfortunately, it is difficult to assess this method’s effectiveness and establish a consensus due to the small number of studies. However, there is still a lack of randomized controlled trials supporting the efficacy of fat and adipose stem cell transplantation in burns.

Additionally, randomized controlled trials on MSCs isolated from bone marrow and adipose tissue are necessary to determine if these therapies are effective. However, such studies are unlikely to be carried out on patients with extensive, deep burns because these burns are rare and usually involve complex clinical decisions using different therapies that may vary between patients. Therefore, randomized trials of patients with smaller burns are recommended, as these burns occur more frequently, and the collection of data from small study groups may be more manageable. Besides, these studies should be performed with the longest possible observation time to assess the long-term safety and efficacy of cell-based therapies. 

Furthermore, other approaches not discussed here seem to have significant therapeutic potential, including epidermal stem cells from hair follicles, embryonic cells, or induced pluripotent stem cells; however, future clinical trials will determine their effectiveness.

## 5. Conclusions

Although much progress has been made to demonstrate cell therapies′ effectiveness in burns, there are still scientific and technical challenges that need to be solved to introduce cell therapies as a standard clinical practice. Further evidence, including clinical trials, as well as studies assessing cell graft take and survival, is needed to demonstrate the clinical efficacy and safety of cell therapies in burns. Furthermore, the cost-effectiveness balance for cell therapy products is also a challenge. Finally, there is a high demand to determine the fate of transplanted cells and the number and type of cells required to obtain the best clinical outcome, as well as the most effective delivery system. We hope that our review will create a basis for further clinical studies and experimental research.

## Figures and Tables

**Table 1:** Clinical applications of autologous keratinocytes, products based on autologous keratinocyte and autologous engineered skin in burns (NA—not available, CEA—cultured epithelial autograft, AT—active treatment, CT—control group, TBSA—total body surface area, CEA/A—cadaver allograft followed by placement of CEA onto an allodermis base, STSG—split-thickness skin graft, TESE—tissue-engineered skin equivalent, CSS—cultured skin substitutes).

Therapy	Compared to	Route and Number of Administrations	Burn Characteristics	Follow Up	# of Patients	Age Range [Years]	Result	Author	Year
**Autologous keratinocytes**									
cultured epithelium	NA	grafts placed on a wound bed	major burns	NA	2	5–6	permanent epidermis generation	Gallico et al. [24]	1984
cultured autologous epidermis	NA	NA	second-and third-degree burn wounds	up to 4 years	17	NA	less hypertrophic scar formation	Teepe et al. [25]	1990
CEA	NA	NA	NA	NA	104	NA	final take rate of about 60%	Odessey et al. [26]	1992
cultured autologous keratinocytes	without graft	grafts	massive burns	NA	64 (22–AT, 42–CT)	NA	mortality rate reduced from 48 to 14%	Munster et al. [27]	1996
sole-derived CEA	Axilla-; groin- or foreskin-derived CEA	grafts	full-thickness burn wounds or giant congenital nevi	3,5 years	12	0–17	re-expression of K9 after grafting on muscle fascia	Compton et al. [28]	1998
CEA/CellSpray/ CEA + CellSpray	NA	application to the wound bed	burns > 50% TBSA	NA	62	30–49	reduction on surgical intervention and total length of stay in hospital	Wood et al. [21]	2006
CEA/A	NA	application to the wound bed	large burns	3–90 months	88	0.5–73	the mean final take rate 72.7%	Sood et al. [22]	2010
CEA	NA	application to the wound bed	burns	NA	63	2–70	good outcome associated with young age and low number of infectious complications	Cirodde et al. [34]	2011
STSG + cultured autologous keratinocytes	cultured autologous keratinocytes alone, STSG alone	application to the wound bed	full-thickness skin burn, 55–65% TBSA	NA	20	4–12	accelerated wound closure, better esthetical results	Chrapusta et al. [30]	2014
CEA	non-CEA	mean 2,1 applications to the wound bed	burns; 60–80% of the TBSA	NA	177 (CEA: 96; non-CEA: 81)	NA	improved survival	Kym et al. [33]	2015
CAE only and CAE after allogeneic cultured epidermis application	NA	grafts, in extensive burns in combination with large meshed STSG (1:6–1:12)	STSG donor sites and deep second-degree burns	NA	63	0.75–58	increased surface of the epidermal barrier	Auxefans et al. [29]	2015
CEA with a wide split auto mesh graft or patch graft	NA	1 application	severe burns	NA	5	13–28	excellent epithelialization	Matsumura et al. [31]	2016
STSG + CEA	micrografting + CEA	grafts	severe burns	NA	STSG + CEA: 10; micrografting + CEA: 14	NA	significantly lower average area amount of skin used in the micrografting group	Chua et al. [35]	2018
CEA + widely meshed STSG (meshing ratio 1:3)/ Cuono method/ CEA only	NA	an average of 8 sheets of CEA	burns ≥ 35% TBSA	12 months	12 (32 sites)	22–67	combination of STSG and CEA increases wound closure and improves CEA take rate	Lo et al. [36]	2019
CEA	NA	NA	burns	NA	954	<1–>80	increased survival rate when combined with STSG	Hickerson et al. [37]	2019
**Products based on autologous keratinocytes**									
Epicel^®^	NA	grafts	burns	NA	30	2.5–70	very high beneficial value in the management of burns > 60% TBSA	Carsin et al. [39]	2000
ReCell^®^	skin grafts	spray	deep partial-thickness burns	6 months	82 (ReCell^®^–42, skin grafting–40)	NA	ReCell^®^gives similar results and is less invasive than skin grafting	Gravante et al. [44]	2007
Keraheal™	NA	spray; combined with 1:4–6 mesh graft	extensive burns	24 weeks	29	30–49	CEA’s take rate 100% after 4 weeks; enhanced take rate of a wide meshed autograft	Yim et al. [41]	2011
Keraheal™	NA	spray; combined with 6:1 mesh graft when burn over 40% TBSA	full-thickness skin wound, TBSA 30–70%	up to 39 months	16	18–70	CEA’s take 68% after 4 weeks, 90.0% after 8 weeks	Lee et al. [13]	2012
Biobrane^®^ with or without ReCell^®^	local standard treatment with surgery at 10 days	application to the wound bed	a pediatric partial-thickness scald injury	until 6 months post-burn	13 (Biobrane^®^ only-4; Biobrane^®^ and ReCell^®^ –5; CT –4)	Biobrane^®^only: 1.5–8.8; Biobrane^®^ and ReCell^®^:0.8–1.8; CT: 2.5–7.1	the best outcome when Biobrane^®^ combined with ReCell^®^; decreased healing time, less pain, and better scar outcomes	Wood et al. [45]	2012
ReCell^®^	STSG	spray	partial-thickness burns	52 weeks	10	NA	decreased donor site size, comparable outcomes with MSTSG treatment	Sood et al. [47]	2015
JACE^®^ CEA	NA	grafts	burns > 30% TBSA	52 weeks	216	0–99	increased survival rate	Matsumura et al. [50]	2016
JACE^®^ CEA on meshed 3:1 split-thickness dermis graft or meshed 6:1 split-thickness autograft	NA	application to the wound bed	massive burns	NA	3	51–66	almost all of the burn wounds had healed at 6 weeks after surgery	Hayashi et al. [48]	2018
**Autologous engineered skin**									
cultured autologous keratinocytes and fibroblasts	NA	grafts	full-thickness burn wounds	4 weeks	4	20–53	advance in the care of extensively burned patients	Hansbrough et al. [51]	1989
autologous TESE (cultured keratinocytes and fibroblasts)	NA	sheet	third-degree burn wounds	up to 9 months	4	29–63	appropriate for permanent repair of full-thickness skin defects	Takami et al. [53]	2004
CSS (autologous cultured keratinocytes and fibroblasts)	STSG	grafts, CSS were meshed at a ratio of 1:1.5 and applied weekly	full-thickness Burns	2 to 7 years in 9 patients	40	0.6–17	better pigmentation, less scaring; no differences in qualitative outcomes after 1 year	Boyce et al. [52]	2006

**Table 2:** Clinical use of allogeneic keratinocytes and fibroblasts in burns (NA—not available, AT—active treatment, CT—control group, DDB—deep partial-thickness burn wounds, STSG—split-thickness skin graft, CDS—cultured dermal substitute).

Therapy	Control/Compared To	Route of Administration	Burn Characteristics	Follow Up	# of Patients	Age Range [Years]	Result	Author	Year
**Allogeneic keratinocytes**									
cultured epidermal cells	NA	sheets	second-degree burn wounds	9 months	3	25–49	accelerated healing and an excellent cosmetic result	Hefton et al. [54]	1983
cultured allogeneic epidermal cells	NA	sheets	the donor site, second-and third-degree burn wounds	NA	26	12–70	poor growth of cells placed on full-thickness, or third-degree burns	Madden et al. [55]	1986
banked cultured human epidermal allografts	NA	grafts	donor sites and DDB	3 months	donor site –10; DDB –10	donor site: 12–41; DDB: 12–32	AT–reepithelialization in about 6.9 days, CT–in an average of 11.1 days.	Rivas-Torres et al. [56]	1996
frozen cultured human allogeneic epidermal sheets	side-by-side with skin donor sites	sheets	deep and superficial partial-thickness burns	NA	11	NA	faster complete re-epithelialization and reduced healing time	Alvarez-Diaz et al. [57]	2000
cryopreserved cultured epidermal allografts	non-grafted areas	grafts	DDB, split-thickness skin donor sites	1–8 years (mean: 4.75 years)	DDB–43, donor site –12	DDB: 4–17; donor sites: 1–17	suppressed scar formation in both DDB and split-thickness skin donor sites	Yanaga et al. [17]	2001
allogeneic keratinocytes	split skin grafts	sheets	deep dermal hand burns	AT–mean 24,8 months; CT–mean 22 months	16 hands–AT, 17 hands–CT	16–80	no statistical significant differences	Haslik et al. [58]	2010
cultured allogeneic keratinocytes	NA	sheets	STSG donor sites and deep second-degree burn wounds	NA	donor sites–59,burns–11	1.8–87	complete healing in 6.4– 7 days.	Auxenfans et al. [14]	2014
**Allogeneic keratinocytes and fibroblasts**									
meshed (1:1.5), unexpanded Apligraf^®^ applied over meshed (≥ 2: 1) expanded autograft	meshed (≥ 2:1) expanded autograft alone or meshed (≥ 2:1) expanded autograft covered by meshed (1.5:1) unexpanded allograf	sheets	burns	24	38	3–78	Apligraf^®^ sites better than the control for 22 (58%) patients	Waymack et al. [62]	2000
OrCel™	dressing Biobrane-L^®^	applied to the site wound	donor sites	6	82	1–88	accelerated wound healing, reduced scarring	Still et al. [65]	2003
Apligraf^®^ or Apligraf^®^ dermis only (without epidermis)	dressings only	sheets	partial-thickness wounds	2	10	37.01–64.59	4-weeks persistence of donor DNA	Hu et al. [63]	2005
**Allogeneic fibroblasts**									
allogeneic CDS overextended auto-skin graft	NA	applied repeatedly at intervals of 5–7 days on the auto-skin graft on the wound	deep burn wounds, necrotizing fasciitis	NA	3	39–88	successful take of mesh auto-skin graft and prompt epithelization	Kashiwa et al. [66]	2004
alloskin over meshed STSG	side-by-side with petroleum jelly–impregnated gauze over meshed STGS	Applied on the wound	third-degree burn wounds	1 year	14	NA	reduced healing time but no difference after 1-year follow up	Moravvej et al. [67]	2012

**Table 3:** Clinical applications of mesenchymal stem cells in burns (CMSCs—cadaveric bone marrow mesenchymal stem cells, NA—not available, AMSG—autologous meshed skin grafting, FMSC—fibroblast-like bone marrow mesenchymal stem cells, SG—skin grafts, BMSCs—bone marrow mesenchymal stem cells, UC-MSCs—umbilical cord blood-derived mesenchymal stem cells, TBSA—total body surface area).

Therapy	Compared To	Route and Number of Administrations	Burn Characteristics	Follow Up	# of Patients	Age Range [Years]	Result	Author	Year
allogeneic FMSC	NA	transplantation on the surface of the wound; followed by SG	extensive skin burn	NA	1	45	promoted and accelerated healing	Rasulov et al. [68]	2005
sweat gland-like cell derived from BMSCs	NA	cells transplanted to the wound, covered with a decellularized allogeneic dermal matrix with numerous laser-punched holes, granulated autologous skin grafting, and allogeneic skin	burn scars devoid of perspiration function	NA	5	7–21	recovery of perspiration function	Sheng et al. [69]	2008
autologous cultured BMSCs + skin graft	skin graft alone	cell injection to the wound	extensive skin burn,	2 years	1	19	smaller risk of contraction of the skin graft	Xu et al. [70]	2012
CMSCs	NA	2 applications of CMSCs followed by AMSG	deep skin burns	3 years	1	26	almost no scar or deformity in the places treated with CMSCs	Mansilla et al. [71]	2015
BMSCs and UC-MSCs	early excision and graft	cell injection to the wound	full thickness burn, 10–25% TBSA	6 months	60	BMSC: 20–27; UC-MSC: 18–29; CT: 18–35	reduced hospitalization time in both BMSC and UC-MSC group	Abo-Elkheir et al. [72]	2017

**Table 4:** Clinical applications of cell-based therapies in radiation burns (BMSCs—bone-marrow mesenchymal stem cells; BMNCs—bone marrow mononuclear cells, ADSCs—adipose-derived stem cells; rh-bFGF—human recombinant fibroblast growth factor; vLPM —lyopreserved placental membrane containing viable cells).

Therapy	Compared To	Route and Number of Administrations	Burn Characteristics	Follow Up	# of Patients	Age Range	Result	Author	Year
autologous lipoaspirates containing ADSCs from a healthy donor site	NA	repeated low-invasive computer-assisted injection in supraclavicular region, the anterior chest wall	progressive lesions after radiation therapy	12, 18 and 31 months	20	37–71	progressive regeneration, including neovessel formation and improved hydration; systematic improvement or remission of symptoms	Rigotti G et al. [77]	2005
autologous BMSCs	NA	subcutaneous and intramuscular administrations	radiation burn	11 months	1	27	healing progression, significant therapeutic improvement	Lataillade et al. [75]	2007
autologous BMMNCs	NA	intradermal administrations	radiation burn	8 months	1	32	progression of the healing process, complete pain collapse	Bey et al. [73]	2010
autologous ADSCs, an angiogenic and mitogenic factor of rh-bFGF, and an artificial dermis	NA	intradermal injections and soaked with the artificial dermis	radiation burn	1.5 years	1	NA	healed wound; the regenerated tissue developed maturely in 1.5 years	Akita et al. [76]	2010
human cadaveric MSC	NA	instilled around and within the lesion	radiation-induced skin lesion	NA	1	66	reduction of the inflammation process, skin quality, and vasculature improvement	Portas et al. [74]	2016
vLPM	NA	allograft; 12 applications	radiation necrosis wound	3 months	1	73	wound closure in 98 days	Regulski et al. [12]	2019

## Data Availability

Not applicable.
